# Lack of T-bet reduces monocytic interleukin-12 formation and accelerates thrombus resolution in deep vein thrombosis

**DOI:** 10.1038/s41598-018-21273-5

**Published:** 2018-02-14

**Authors:** Tanja Schönfelder, Moritz Brandt, Sabine Kossmann, Tanja Knopp, Thomas Münzel, Ulrich Walter, Susanne H. Karbach, Philip Wenzel

**Affiliations:** 1 Center for Thrombosis and Hemostasis Mainz, Mainz, Germany; 2 Center for Cardiology—Cardiology I, Mainz, Germany; 3 Deutsches Zentrum für Herzkreislaufforschung (DZHK)—Partner site Rhine-Main, University Medical Center Mainz, Langenbeckstrasse 1, 55131 Mainz, Germany

## Abstract

The role of leukocytes in deep vein thrombosis (DVT) resolution is incompletely understood. We determined how depletion of lysozyme positive (LysM^+^) cells and a switched-off type 1 immune response influences thrombus resolution. DVT was induced in 12-week-old male mice by inferior vena cava (IVC) stenosis. Toxin mediated depletion of myeloid cells improved thrombus resolution in mice with Cre-inducible expression of the diphtheria toxin receptor in LysM^+^ cells. This correlated with decreased CD45^+^ cells, a population shift of Gr-1^+^ to Gr-1^−^ CD11b^+^ myelomonocytic cells (flow cytometry) and an increase in CC-chemokine ligand 2, interleukin-4 and interleukin-10 mRNA expressions. Tbx21^−/−^ mice (lacking transcription factor T-bet and marked by an attenuated type 1 immune response) with DVT had faster thrombus resolution, a reduction of pro-inflammatory Ly6C^hi^ monocytes in thrombi and decreased interleukin-12p40 mRNA expression than control mice resulting in increased vascular endothelial growth factor mRNA expression and improved neovascularization of thrombotic veins. Transfer of Tbx21^−/−^ bone marrow into irradiated Tbx21^+/+^ recipients lead to accelerated thrombus resolution with lower T-bet-dependent interleukin-12p40 mRNA levels following IVC-stenosis. We conclude that inhibition of Tbet^+^ interleukin-12 forming myelomonocytic cells accelerated thrombus resolution. Modulating the inflammatory immune response might be an approach to improve therapy of DVT.

## Introduction

Deep vein thrombosis (DVT) with its sequel venous thromboembolism (VTE) is one of the leading causes of morbidity and mortality worldwide with an annual incidence of up to 600,000 in the US alone^1^. It is more and more appreciated, that components of the cellular and humoral immune response, but also platelets^2,3^ and complement factors^4^ contribute to DVT initiation and propagation. In addition, an orchestrated immune response involving precise modulation of cytokine signaling (e.g. interferon gamma, Ifnγ^5^) and cellular responses of T-cell receptor beta^+^ CD4^+^ as well as CD8^+^ and effector memory (T_EM_) T-cells^6^ seems to impact on thrombus resolution. A decisive role in both the initiation and the resolution of DVT is played by myelomonocytic cells (macrophages, monocytes and neutrophil granulocytes)^7–9^. Clinical data suggest, that monocytosis is associated with DVT^10^ and that avoidance of monocyte adhesion and activation might prevent thrombosis^11^. In mouse models of DVT, flow reduction in the inferior vena cava leads to secretion of von Willebrand factor from activated endothelial cells, which is a carrier of coagulation factor VIII but also captures platelets via glycoprotein Ib alpha binding. When this platelet binding is interrupted, endothelial recruitment of leukocytes through P-selectin mediated signaling is drastically reduced and DVT formation is blunted^8,12^. Myelomonocytic cells are a rich source of tissue factor, a major initiator of thrombin generation *in vivo*^13^. Mice are largely protected from DVT initiation when lysozyme M (LysM) positive monocytes are tissue factor (TF)-deficient. The interplay of TF-bearing LysM^+^ cells and platelets is required to provide a pro-coagulant microenvironment, enabling contact phase factors like FXII and FXI and to propagate and amplify DVT formation^3,8^.

A sequential alternation of predominantly myeloid immune cells with humoral activity (and to a lesser extent lymphocytes) in the vein wall within the first week post DVT has been shown in the stasis model of DVT in rats^9^. In particular, monocytes can play a major role in thrombus resolution directed at least in part by the monocyte chemoattractant protein-1 (MCP1)/CC-chemokine receptor 2 (CCR2) axis^14^. For example, DVT resolution was delayed, when neutrophil and monocyte chemotaxis into the thrombus was blocked by an anti-CXC-motif chemokine receptor 2 (CXCR2)-antibody or in CXCR2^−/−^ mice^15^, and mice lacking CCR2, the receptor for MCP1 had decreased monocyte influx and impaired thrombus resolution^16^. It was described that increasing macrophage numbers in venous thrombus in rats enhanced thrombus resolution and that treatment with MCP1 increased thrombus resolution by stimulating recanalization, paralleled by an increase of ED1^+^ monocytes in the vein wall adjacent to the thrombus^17^. It was shown that fewer CCR2^+^ monocytes/macrophages were present in venous thrombi of mice lacking toll like receptor 9 compared to control mice going along with larger venous thrombi at day 8 and day 21 after DVT induction^18^. Likewise, thrombus resolution is accelerated by F4/80^+^ myelomonocytic cells secreting matrix metalloproteinase 9, which is blocked in an Ifnγ-dependent fashion^5^. We have recently shown, that Ifnγ released by T box expressed in T-cells (T-bet)^+^ natural killer (NK) cells can activate LysM^+^ positive myelomonocytic cells to form more interleukin (IL)-12, which in turn require T-bet expression to release IL-12, to stimulate NK cells^19^ and to perpetuate vascular dysfunction in arterial hypertension^20^. In a stenosis model of DVT, a cocktail of cytokines typically released by myelomonocytic cells including IL-12 and IL-18 fosters Ifnγ-production by T_EM_ cells, and deficiency of these Ifnγ-producing cells reduces infiltration of lymphocyte antigen 6 complex locus C1 (Ly6C)^hi^ inflammatory monocytes into thrombotic veins and improves thrombus resolution^6^. Interestingly, polarization of myeloid cells/macrophages towards a more M1 like phenotype (classically activated, pro-inflammatory) also retarded thrombus resolution in a stasis model of DVT^21^.

These data indicate, that immunomodulation of monocyte and/or macrophage phenotype and function would be required to take advantage of the capacity of these cells to improve thrombus resolution without impacting on thrombus generation. We therefore set out to test the hypothesis, that depletion of LysM^+^ monocytes would accelerate thrombus resolution and that switching off a type 1 inflammatory response with its signature cytokine Ifnγ as suggested by previous work^5,6,20,21^ might be equally efficient. As murine DVT model we use the subtotal IVC ligation model (IVC stenosis model)^4,8,12,22^ which is regarded to be closest to the physiological conditions in human thrombus formation resulting from partial stasis in blood flow leading to DVT.

## Results

### Sequential recruitment of inflammatory cells to the venous thrombus following vena cava inferior stenosis in mice

**Figure 1:**
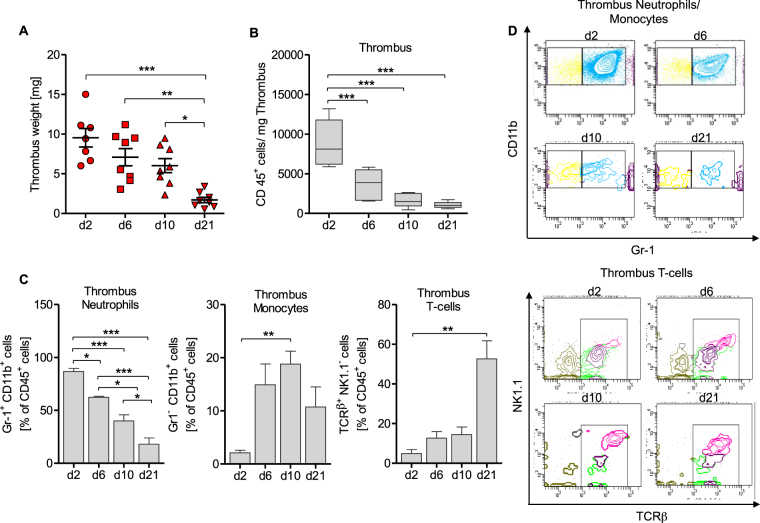
The distribution of inflammatory cells changes in the resolution process. In the IVC-stenosis model thrombus weight decreases during a time period of 21 days (**A**) as well as the total cell count of CD45^+^ cells/mg thrombus (**B**). Results are shown as median with interquartile range, n = 5 to 6 animals per group. One way-ANOVA/Bonferroni’s Multiple Comparison test; *p ≤ 0.05; **p ≤ 0.01; ***p ≤ 0.001. The flow-cytometric analysis of CD45^+^CD11b^+^Gr-1^+^ neutrophils, CD45^+^/CD11b^+^/Gr-1^−^ monocytes and CD45^+^/CD11b^−^/TCRβ^+^ T-cells in thrombus tissue reveals different peaks of these cells during a time course of day 2, 6, 10 and 21 (**C**). Results are shown as mean ± SEM, n = 5 to 6 animals per group. One way-ANOVA/Bonferroni’s Multiple Comparison test; *p ≤ 0.05; **p ≤ 0.01; ***p ≤ 0.001. (**D**) Representative FACS-plots of CD11b^+^/Gr-1^+^ neutrophils (blue), CD11b^+^/Gr-1^−^ monocytes (yellow) and NK1.1^−^/TCR beta^+^ T-cells in thrombus material (day 2, 6, 10, 21 post subtotal ligation) of C57BL/6 mice.

Thrombus size reached a maximum 2d after subtotal inferior vena cava inferior (IVC) ligation (IVC stenosis model). Thrombus mass reduced gradually during the ensuing 21 days (Fig. 1A). Reduction in thrombus mass was paralleled by a decrease of the absolute number of CD45^+^ leukocytes per mg thrombus (Fig. 1B). To investigate relative distribution patterns of myeloid and lymphoid cells within that population of CD45^+^ leukocytes, we performed flow cytometric analysis of venous thrombi. In this population, the proportion of Gr-1^+^CD11b^+^ immune cells consisting mainly of neutrophils and inflammatory monocytes gradually declined within these 21d. The proportion of Gr-1^−^CD11b^+^ monocytes peaked at d10 post thrombus initiation and declined after that. In contrast, the fraction of TCRβ^+^ T-cells was <10% at d2, still below 20% at d10, and peaked at d21 post IVC stenosis (Fig. 1C,D). The absolute number of CD45^+^ leukocytes within the thrombus was only 1018 (568–1733) cells/mg on d21 compared to 8110 (5893–13186) cells/mg on d2 (median with IQR, Fig. 1A), implying that the number of T-cells was generally lower and less dynamically regulated in DVT resolution within 21d post IVC stenosis. In this regards, our flow cytometry data suggest a possibly important role of innate myelomonocytic cells inside the thrombus in DVT resolution.

### Depletion of Lysozyme M^+^ myelomonocytic cells accelerates thrombus resolution

**Figure 2:**
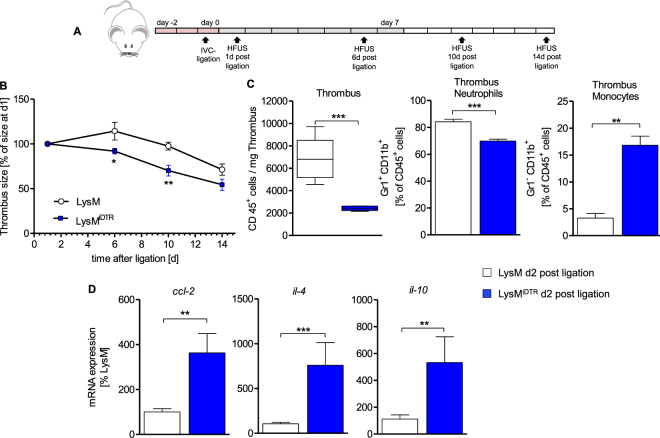
Depletion of Lysozyme M positive cells leads to faster thrombus resolution in IVC ligated mice. (**A**) Scheme of experimental setup. US, ultrasound. Red boxes, DTX high dose. Grey boxes, DTX low dose. Each box represents 1day. (**B**) Sizes determined by high frequency ultrasound calculated as percentage at day 1 after subtotal ligation, of thrombi in LysM and LysM^iDTR^ group at indicated time points after subtotal ligation. Mean ± SEM; two-way ANOVA/Bonferroni post hoc test; n = 8; *p ≤ 0.05; **p ≤ 0.01. (**C**) Flow cytometric analysis of cell count of CD45^+^ cells/mg thrombus and neutrophils and monocytes displayed as percentage of CD45^+^ cells (day 2 post subtotal ligation) in thrombus tissue of LysM^iDTR^ mice compared to LysM controls. (**D**) mRNA expression levels of *ccl-2*, *il-4* and *il-10* in thrombus material. Mean ± SEM; t-test; n = 5–8; *p ≤ 0.05; **p ≤ 0.01; ***p ≤ 0.001. HFUS, high frequency ultrasound.

Using Cre-inducible diphtheria-toxin mediated depletion of LysM^+^ cells (Fig. 2A: scheme of depletion regimen), we observed a significantly accelerated resolution of DVT in the IVC ligated LysM^iDTR^ mice as compared to LysM controls (Fig. 2B). Investigating the thrombus composition at d2 post IVC ligation, flow cytometry analysis revealed a significantly reduced absolute number of CD45^+^ cells (Fig. 2C), a significant reduction of the relative percentage of CD11b^+^Gr-1^+^ inflammatory myeloid cells as well as a significant increase of the relative percentage of CD11b^+^Gr-1^−^ monocytes (Fig. 2C) in the IVC ligated LysM^iDTR^ as compared to controls. In parallel, mRNA expression of *ccl-2* (encoding for CC-chemokine ligand 2/MCP1), *il-4* and *il-10* was significantly increased in LysM^iDTR^ mice compared to controls (Fig. 2D). These data suggest, that depletion of LysM^+^ cells in the described manner (Fig. 2A) accelerates thrombus resolution paralleled by a dampened pro-inflammatory microenvironment and skews the immune response towards a “Th2 type” cytokine signature inside the thrombus.

### The use of a mouse model with a switched-off type 1 immune response reveals improved thrombus resolution

**Figure 3:**
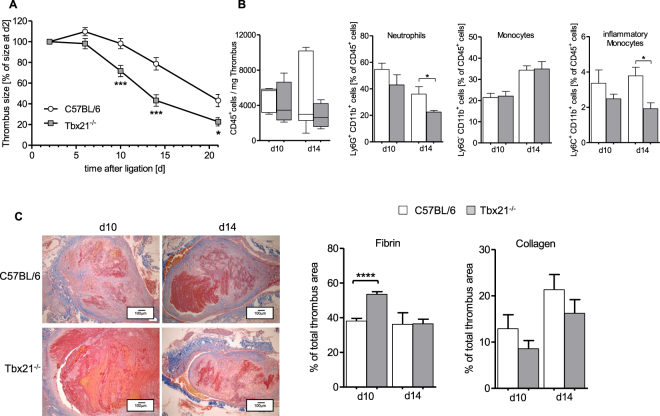
Increased thrombus resolution in the Tbx21^−/−^ mice. (**A**) Thrombus sizes determined by high frequency ultrasound (HFSU) calculated as percentage of the size at d2 in C57BL/6 and Tbx21^−/−^ group at indicated time points following IVC stenosis. Mean ± SEM; two-way ANOVA/Bonferroni post hoc test; n = 7; *p ≤ 0.05; ***p ≤ 0.001. (**B**) Flow cytometric identification of thrombus inflammatory cells at indicated days after subtotal ligation of control compared to Tbx21^−/−^ mice. Data shown as median with interquartile range and mean ± SEM; t-test; n = 4–5; *p ≤ 0.05. (**C**) Left panel: Histological staining (MTC staining) of IVCs after IVC stenosis at indicated time points, representative pictures are shown. Magnification x10. Right panel: Summary of the quantitative analysis on fibrin and collagen contents of venous thrombi in 4 mice per group for MTC staining. Mean ± SEM; t-test; n = 4–5; ****p ≤ 0.0001.

**Figure 4:**
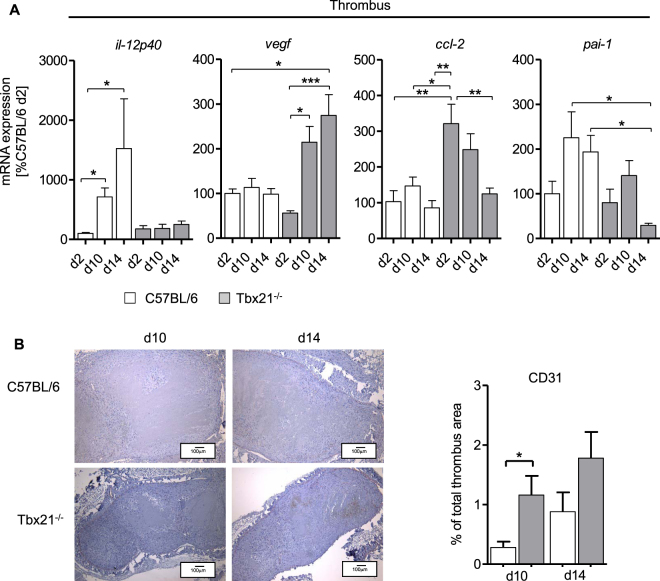
Enhanced recanalization of thrombus material and altered mRNA expression patterns in Tbx21^−/−^ mice. (**A**) Analysis of *il-12p40*, *vegf*, *ccl-2* and *pai-1* mRNA expression in thrombus material of control compared to Tbx21^−/−^ mice. Mean ± SEM of n = 4–7 animals per group; Kruskal-Wallis-test with Dunn’s Multiple Comparison test (for *il-12p40*, *vegf*) and one-way ANOVA with Bonferroni’s Multiple Comparison test (*ccl-2* and *pai-1*); *p ≤ 0.05; **p ≤ 0.01; ***p ≤ 0.001. (**B**) Immunohistochemistry (CD31 staining) of IVCs following subtotal ligation at indicated time points. Summary of the quantitative analysis, results in mean ± SEM n = 3–4 animals per group; t-test; *p ≤ 0.05.

T-bet (encoded by Tbx21) is essential to govern the transcription of Ifnγ, the signature cytokine of the type 1 immune response in lymphoid cells (mainly T-cells and NK cells)^23^ and IL-12 in monocytes^19^. Tbx21^−/−^ mice are therefore characterized by genetically skewing towards a “Th2-type” immune response. Thrombus resolution was significantly accelerated in Tbx21^−/−^ mice compared to controls (Fig. 3A), resembling the findings in the LysM^+^ cell-depleted mice (Fig. 2B). Flow cytometry analysis of the cellular composition of the thrombi revealed comparable absolute numbers of CD45^+^ leukocytes per mg thrombus with significantly reduced percentage of lymphocyte antigen 6 complex locus G (Ly6G)^+^ neutrophils and inflammatory Ly6C^hi^ monocytes in Tbx21^−/−^ compared to controls at d14 post IVC stenosis (Fig. 3B). Histological examination of the thrombotic tissue showed significantly higher fibrin content in in Tbx21^−/−^ compared to controls at d10 and significantly reduced collagen content in the thrombi of Tbx21^−/−^ at all time points analyzed (Fig. 3B,C). We next analyzed the change of mRNA expression within thrombotic tissue in the resolution process at d2, d10 and d14 post IVC stenosis. Expression of *il-12p40* mRNA gradually and significantly increased in Tbx21^+/+^ whereas this rise was completely absent in Tbx21^−/−^ mice, consistent with the switched-off T-bet-dependent inflammatory immune response in myeloid cells (Fig. 4A). Vice versa, Tbx21^−/−^ mice showed a steady increase of *vegf* mRNA expression encoding for vascular endothelial growth factor over a period of 14d, which was not detectable in Tbx21^+/+^ mice. Expression of *ccl-2* mRNA was significantly increased mainly on day 2 and slightly declined after that over the period of 14 days in Tbx21^−/−^ mice, while this temporal regulation of mRNA expression was not observed in wild type controls. Interestingly, the expression of *pai-1* mRNA encoding for plasminogen activator inhibitor-1 (PAI-1), the main inhibitor of plasmin formation antagonizing thrombus resolution was reduced in the Tbx21^−/−^ mice compared to controls, compatible with accelerated thrombus resolution.

The altered mRNA expression profile in Tbx21^−/−^ mice favored attenuated inflammation on the one hand and improved revascularization and thrombus resolution as well as attraction of reparative monocytes on the other hand. This was mirrored by the histological analysis the thrombotic material: Compared to controls, Tbx21^−/−^ mice had a higher content of CD31^+^ endothelial cells in the resolution phase post IVC subtotal ligation (Fig. 4B). Taken together, these data indicate that the switched-off type 1 inflammatory response in the Tbx21^−/−^ mice translates into better re-endothelialisation of thrombus material and faster thrombus resolution.

### T-bet dependent impact on thrombus resolution is mediated by bone marrow-derived cells

**Figure 5:**
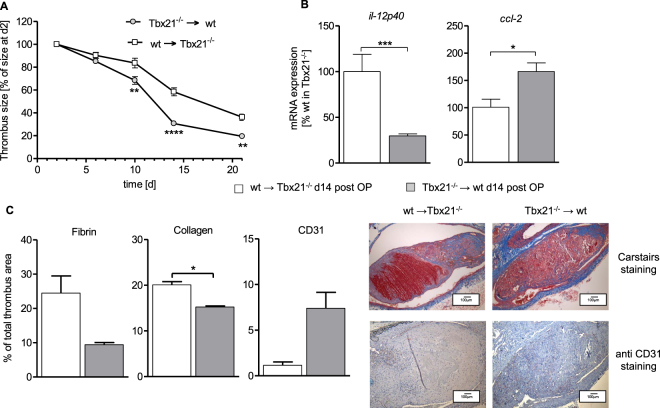
Critical role of T-bet in bone marrow-derived cells for thrombus resolution. (**A**) Thrombus sizes determined by high frequency ultrasound calculated as percentage at day 2, of thrombi in irradiated C57BL/6 wild type mice (CD45.1^+^ Ly5.1) mice transplanted with bone marrow of Tbx21^−/−^ mice (Tbx21^−/−^ → wt) compared to irradiated Tbx21^−/−^ mice transplanted with wild type bone marrow (wt→ Tbx21^−/−^ mice) at indicated time points following IVC stenosis. Mean ± SEM; two-way ANOVA/Bonferroni post hoc test; n = 3–4; **p ≤ 0.01; ****p ≤ 0.0001. (**B**) Analysis of *il-12p40* and *ccl-2* mRNA expression in thrombus material. Mean ± SEM. n = 3–4 animals per group; t-test; *p ≤ 0.05. (**C**) Histological staining (Carstairs staining) and immunohistochemistry (CD31 staining) of IVCs 14d after subtotal ligation. Magnification x10. Summary of the quantitative analysis, results in mean ± SEM. n = 3–4 animals per group; t-test; *p ≤ 0.05.

To address, whether the observed effects are mediated by bone marrow-derived cells, we irradiated control mice and transplanted them with bone marrow cells collected from Tbx21^−/−^ mice and vice versa. Resulting chimeras (Tbx21^−/−^ → wt and wt → Tbx21^−/−^) were subjected to subtotal IVC ligation, followed up by high frequency ultrasound for 21d and tissue collection and analysis at d21. Compared to wt → Tbx21^−/−^ mice, Tbx21^−/−^ → wt chimeras had significantly accelerated thrombus resolution (Fig. 5A), attenuated *il-12p40* and higher *ccl-2* mRNA expression (Fig. 5B), decreased fibrin and collagen content as well as increased CD31^+^ endothelial cells (Fig. 5C) inside the thrombus. Therefore, the Tbx21^−/−^ → wt chimera largely reproduced the phenotype of Tbx21^−/−^ mice, indicating that bone-marrow derived cells are the mediators of the T-bet dependent impact on thrombus resolution in DVT.

## Discussion

In the current work we show that DVT in the subtotal IVC ligation model (IVC stenosis) is hallmarked by a time-dependent sequence of infiltrating immune cells that accumulate and diminish inside the thrombus during thrombus resolution. Toxin-mediated selective depletion of LysM^+^ cells in transgenic LysM^iDTR^ mice accelerated thrombus resolution accompanied by a reduction of inflammatory cells inside the thrombus. In a more subtle genetic approach, we were able to show that expression of the transcription factor T-bet in bone marrow derived cells directed IL-12 formation and accumulation of Ly6C^hi^ inflammatory monocytes inside the thrombus following IVC stenosis. Deletion of T-bet reduced this, and in consequence caused enhanced expression of VEGF with improved reendothelialization of the thrombus and faster resolution of the DVT.

Our findings in principle confirm earlier reports using histological methods on immune cell distribution in the vein wall of rats in the stasis model of DVT^9^. Combination of the morphometric analysis of microscopically visible cells with cytokine/chemokine expression patterns (increase of the chemokines MCP1 and CXCL5 and the type 1 immune response genes tumor necrosis factor alpha and interleukin 6) identified a sequence of neutrophils, monocytes/macrophages and “thrombus organizing” myeloid cells with humoral activity predominantly within the first week post DVT induction^9^.

In analogy to this study and to the findings published earlier in mouse models of myocardial infarction (MI), we present here the first study demonstrating the time dependent three-phasic infiltration of leukocytes into the thrombus in DVT resolution based on flow cytometric analysis: First, neutrophils and inflammatory monocytes, followed by reparative monocytes^24^, and then by a persistence of T-cells^25^. Similarly, in the work of Nosaka *et al*. immunohistochemistry of thrombus material had identified a peak of MPO^+^ neutrophils d1 post DVT gradually declining until d5, and a peak of F4/80^+^ myelomonocytic cells at d5 to 10 post DVT, declining until d14^5^. These cell types have been assigned specific roles for both cardiac wounding and healing in MI^24–26^. In the case of DVT, a clear-cut role of myelomonocytic cells has been studied in more detail only in initiation of thrombosis: Depletion of neutrophils and monocytes prevents initiation of DVT, and mice with TF-deficient monocytes are largely protected from DVT development^4,8^. So far, there has only been indirect evidence that monocytes are critical for thrombus resolution: Impaired CCR-2/CCL-2 signaling retards thrombus resolution because it impairs monocyte recruitment^16^, and Ifnγ-deficiency accelerates thrombus resolution because it allows monocytes to secrete more matrix metalloproteinase 9^5^. Lack of this proteinase was described to impair murine thrombus resolution and resulted in a reduced macrophage infiltration and a reduced collagen content in the thrombus^27^. To the contrary, increasing macrophage numbers in venous thrombus were shown to enhance thrombus resolution in rats^14^.

However, thrombus resolution seems to be more complex based on the balanced interplay of myelomonocytic cells: We here provide unequivocal evidence for the role of inflammatory LysM^+^ myeloid cells for thrombus resolution using a direct genetic approach. Depletion of LysM^+^ cells *early* after DVT induction (see Fig. 2A) preferentially deletes the more pro-inflammatory CD11b^+^Gr-1^+^ cells comprising both Ly6G^+^ neutrophils and Ly6C^hi^ monocytes which are a prime source of IL-12, thereby allowing the reparative Gr-1^−^ monocytes to invade and clear the clot.

The LysM^iDTR^ model has been widely used to study the impact of inflammatory myelomonocytic cells, for instance on wound healing^28^, angiotensin II-induced arterial hypertension and vascular dysfunction^29^, cardiac remodeling after MI^30^ and myofibroblast trans-differentiation in renal fibrosis^31^, processes that critically depend on the presence of myeloid cells. The feasibility of the approach was confirmed in our current study, since the toxin-mediated depletion of myelomonocytic cells was effective and sufficient to speed up thrombus resolution.

However, continued and extensive depletion of myeloid cells will increase the susceptibility to potentially lethal infections and disturbs wound healing in a life-threatening way, especially after MI^30^. Depletion of monocytes is therefore not a useful way to translate the findings to therapeutic applicability, and we employed a mouse model that lacks the transcription factor T-bet. We have previously shown, that T-bet deficient Tbx21^−/−^ mice are protected from angiotensin II-induced vascular dysfunction, mainly due to the inability of T-bet negative monocytes to produce the inflammatory cytokine IL-12. This shortage of IL-12 interrupted the positive feedback loop with Ifnγ-forming lymphocytes and resulted in less vascular infiltration of inflammatory monocytes^20^. Indeed, by using the Tbx21^−/−^ mice, we attenuated IL-12 and increased CCL-2 formation, resulting in less abundance of Ly6C^hi^ monocytes in the thrombus.

Mechanistically, we were able to show that expression of PAI-1 is reduced in T-bet deficient mice. PAI-1 deficiency resulted in an increase in thrombus resolution as described previously^32^. It has been shown, that the equilibrated regulation of PAI-1 expression levels which is essential for fibrin deposition and removal is directed by Ifnγ^33^, the signature cytokine of type 1 immune response. Interestingly, it has been shown that Ifnγ is able to inhibit VEGF expression not only in endometrial stromal cells^34^, but also in fibroblasts^35^ and vascular smooth muscle cells^36^. Since Ifnγ-transcription critically depends on T-bet^23^, our finding directly links the switched-off type 1 immune response in Tbx21^−/−^ mice to better thrombus revascularization and resolution in DVT mice.

Recent findings indicate the existence of a specialized population of arterial macrophages, that evolves in part from bone marrow independent precursors, but can be replenished by circulating monocytes. These tissue-specific resident myelomonocytic cells are able to mount the full array of immune responses, presumably including IL-12 formation^37^. It is unknown, however, whether also veins host a subpopulation of resident immune-competent cells that could elicit an inflammatory response including chemokine and cytokine secretion. Secondly, Ifnγ has been shown to impede angiogenesis in development^38^, which might impact on DVT resolution especially in the constitutive knockout model. To control for Ifnγ-dependent effects on developmental angiogenesis and to pinpoint whether the T-bet related effects on thrombus resolution depend on bone marrow-derived cells or putative vascular resident cells, we generated the respective chimeras. The Tbx21^−/−^ → wt chimeras pheno-copied the Tbx21^−/−^ mice, strongly suggesting that lack of T-bet in bone marrow derived myeloid cells results in reduced IL-12 formation and attenuated Ly6C^hi^ monocytosis. In concert with decreased PAI-1 formation and increased VEGF formation directly linked to Ifnγ^33,36^ this caused faster thrombus revascularization and resolution.

Delayed thrombus resolution is a clinical challenge that increases the risk of DVT recurrence and of post thrombotic syndrome, two conditions associated with high morbidity and increased risk of death^39^. Also pulmonary embolism^40^ and chronic thromboembolic pulmonary hypertension^41,42^ are characterized by strong thrombo-inflammatory responses and are potentially life threatening diseases, in which the inflammatory burden correlates with adverse outcomes. In the arena of atherosclerosis, coronary heart disease and atherothrombosis, the interrelationship between inflammation and adverse outcomes has been studied in much more detail^43^. Post MI, a sequential recruitment of immune cells to the infarcted tissue orchestrates wound healing, repair and scar formation^44^, with some similarities to resolution of DVT. Recent evidence from clinical trials suggests, that an efficient anti-inflammatory pharmacotherapy might reduce mortality in secondary prevention in patients with coronary artery disease and high inflammatory burden^45^. Based on these considerations and our results, we conclude that more research is needed to increase our understanding about the type 1 immune response early after initiation of thrombus formation. This insight might prove useful to develop new approaches to speed up thrombus resolution in experimental models, but also in humans suffering from DVT and VTE.

## Materials and Methods

All methods were carried out were in accordance with the Declaration of Helsinki and National Institutes of Health guidelines. All experimental protocols were approved by the Ethics Committee of the University Hospital Mainz and by the Institutional Animal Care and Use Committee (IACUC; Landesuntersuchungsamt Rheinland-Pfalz, Koblenz, Germany).

### Animals

C57BL/6 mice originally obtained from Jackson Laboratories (C57BL/6J; Bar Harbor, USA) as well as Tbx21^−/− ^^46^, LysM^Cre/Cre ^^47^, ROSA26^iDTR/iDTR ^^48^ (crossed to generate male LysM^Cre/wt^ and LysM^Cre/wt^/ROSA26^iDTR/wt^, abbreviated LysM and LysM^iDTR^) all on the C57BL/6J background were used as experimental animals. In the depletion protocol LysM and LysM^iDTR^ received i.p. injections with diphtheria toxin (DTX, Sigma-Aldrich) once daily (solved in PBS; 25 ng/g from day 1–3, 5 ng/g thereafter)^29^. All mice were bred and housed in the Central Laboratory Animal Facility of the University of Mainz (Mainz, Germany).

### Mouse model of subtotal flow restriction in the vena cava inferior

Male animals of at least 12 weeks of age with a minimum body weight of 25 g were anesthetized by intraperitoneal injection of a solution of midazolame (5 mg/kg; Ratiopharm GmbH, Ulm, Germany), medetomidine (0.5 mg/kg body; Pfizer Deutschland GmbH, Berlin, Germany), and fentanyl (0.05 mg/kg; Janssen-Cilag GmbH, Neuss, Germany). The animals were fixed on a custom built-stage and maintained at physiological temperature. Animals were then depilated with hair removal cream at the area of surgery. IVC stenosis was performed as described^22,49^ and introduced by Brill *et al*.^12^. A median laparotomy was performed and the vena cava inferior (IVC) was exposed by atraumatic surgery. We positioned a space holder (Asahi Fielder XT Guide Wire 0.014” [0.36 mm]; Abbot Vascular, Abbot Park, USA) on the outside of the vessel and placed a permanent narrowing ligature (7.0 monofil polypropylene filament, Prolene; Braun, Melsungen Germany) exactly below the left renal vein. Subsequently, the wire was removed to avoid complete vessel occlusion. Side branches were left open in all groups as described previously and mice with side branches closer than 1.5 mm to the site of ligation were excluded from the analysis, resulting in evidence of venous thrombus in 75% of all ligated mice included in the analysis^22^. To rule out endothelial injury as a trigger for venous thrombosis, all mice with bleedings or any injury of the IVC during surgery were excluded from further analysis. Median laparotomy was immediately closed by a 7.0 polypropylene suture (Ethicon). After the surgical procedure, the animals were administered atipamezole (0.05 mg/kg) and flumazenil (0.01 mg/kg) subcutaneously to antagonize anesthesia, postoperative analgesia was carried out with buprenorphine (0.075 mg/kg). For weight and length measurement of thrombi, animals were sacrificed 2, 6, 10, 14 and 21 days after surgery, the IVC was excised below the renal veins and the thrombus was exteriorized.

### Generation of BM chimera

Bone marrow chimeras were generated by lethal irradiation of recipient C57BL/6 Ly5.1 or Tbx21^−/−^ mice with 9.5 Gy from Cs^137^ source (OB58-BA; Buchler, Braunschweig, Germany) and i.v. transfer of 5 × 10^6^ bone marrow cells harvested from femurs and tibias from animals of the indicated donor strain within 24 hours. One week before irradiation and during the first 2 weeks after transplantation, mice were given Borgal antibiotic (Hoechst Roussel Vet, Wiesbaden, Germany) in the drinking water. Mice were rested 7–8 weeks before use in experiments. Blood, aortic and bone marrow chimerism was verified by flow cytometric analysis using CD45.1 (A20, BD Biosciences, San Jose, CA) and CD45.2 (104, eBioscience, San Diego, CA).

Two experimental groups were included in the study: C57BL/6 Ly5.1 wt mice transplanted with Tbx21^−/−^ BM (Tbx21^−/−^→wt) and Tbx21^−/−^ mice reconstituted with Ly5.1 BM (wt→Tbx21^−/−^).

### High frequency ultrasound

Both size and resolution was monitored by high frequency ultrasound, which can be achieved with high sensitivity and accuracy^22,49^ after thrombus development. Anesthesia of mice was induced in a chamber (2–4% isoflurane mixed with 0.2 L/min 100% O_2_) and maintained with a face mask (0.5–1.5% isoflurane with 0.05–0.1 L/min 100% O_2_). Animals were kept on a heated table mounted on a rail system (Visual Sonics, Toronto, Canada). Ultrasound was performed with the Vevo 770 System and a 40 MHz mouse scan head (RMV 706; VisualSonics). Body temperature was monitored using a rectal probe and maintained at 37 °C. The abdomen of the mouse was depilated and warm ultrasound transmission gel was applied to enable visualization and optimize image quality. First, a long axis view was used to visualize the IVC, the stenosis and the formed thrombus. An optimal freeze-frame image was taken manually and, using the Vevo 770 software, the cross-sectional area of the clot was traced to obtain the measurement. The length, width and area of clots were measured applying B-mode.

### Fluorescence activated cell-sorting (FACS) analysis of immune cells

The immunological phenotype of the mice was examined by flow cytometric analysis of TCRß^+^ lymphoid cells (T-cells), as well as CD11b^+^, Gr-1^+^ (Ly6G^+^, Ly6C^+^) and F4/80^+^ myeloid cells (representing neutrophils and monocytes/macrophages) and fixable viability dye (dead cell marker, all from eBioscience, San Diego, CA) in the whole blood, vein and thrombus. Blood erythrocytes were hemolysed by BD FACS lysing solution. The thrombus tissue was mechanically homogenized using a cell strainer of 70 µm. To block non-specific Fc-receptor-mediated binding sites, cells are pre-incubated with unlabeled antibody against CD16/CD32 for 10 minutes (Fc-block). Stained cells were immediately analyzed on a FACSCanto II flow cytometer with FACSDiva software, version 6 (Becton Dickinson and Company, Cockeysville, MD, USA).

### Quantitative RT-PCR

For isolation of venous and thrombus RNA from snap-frozen mouse veins and thrombus material were homogenized with stainless steel micro-pestles (A. Hartenstein, Würzburg, Germany) and for RNA isolation the modified guanidine isothiocyanate method of Chomczynski and Sacchi^50^ was used. RT-PCR was performed with the CFX96 Real-Time PCR Detection System (BioRad, Munich, Germany). For RT-PCR analysis 0.125 µg of total RNA were used with the QuantiTect Probe RT-PCR kit (Qiagen, Hilden, Germany). TaqMan Gene Expression assays were used as probe and primer sets (Applied Biosystems, Foster City, CA) for beta actin (mouse: Mm00607939_s1), TATA-box binding protein (Tbp, mouse: Mm00446973_m-1), MCP-1 (Ccl2, mouse: Mm00441242_m1), IL-4 (mouse: Mm00445259_m1), IL-10 (mouse: Mm00439614_m1), IL-12b (mouse: Mm00434174_m1), Plasminogen activator inhibitor-1 (PAI-1, mouse: Mm00435858_m1). Results were quantified with the relative Ct method and normalized to TATA box binding protein (veins) and beta actin (thrombus) as the endogenous control.

### Immunohistochemistry

Sections were de-paraffinized and blocked in 3% H_2_O_2_ (in methanol) for peroxidase activity. For heat induced epitope retrieval 0.01 M citrate buffer was used. Blocking of unspecific antigen binding sites was performed with 10% normal serum (in PBS). Endothelial cells were visualized using a monoclonal antibody against murine CD31 (1:20) followed by incubation with a peroxidase-conjugated secondary antibody, avidin biotin link (VectorLabs) and peroxidase substrate (AEC; VectorLabs). Sections were briefly counterstained with Gill’s hematoxylin and mounted with ImmoMount (ThermoScientific). For evaluation of fibrin and collagen contents in thrombus material a Carstair’s staining was performed. To distinguish between the cells and the connective tissue the Masson Trichrome staining was performed as described previously^51^. All pictures were recorded with a Olympus BX51 microscope and were quantified by morphometric analyses using image analysis software (Image ProPlus, version 7.0; Media Cybernetics).

### Statistics

Data are expressed as mean ± SEM. Statistical calculations were performed with GraphPad Prism 5 (GraphPad Software Inc, San Diego, CA). D’Agostino-and-Pearson normality test was first performed, and Pearson’s correlation, Fisher’s exact test, Mann-Whitney test, paired or unpaired t-test, Kruskal-Wallis test, one-way-ANOVA or two-way-ANOVA with posthoc Bonferroni’s or Dunn’s Multiple Comparison test were used as appropriate. Values of *p* < 0.05 were considered significant, marked by asterisks: *p < 0.05; **p < 0.01; ***p < 0.001.

## References

[1] Mozaffarian D (2016). Heart Disease and Stroke Statistics-2016 Update: A Report From the American Heart Association. Circulation.

[2] Mackman N (2012). New insights into the mechanisms of venous thrombosis. J Clin Invest.

[3] Stark K (2016). Disulfide HMGB1 derived from platelets coordinates venous thrombosis in mice. Blood.

[4] Subramaniam S (2017). Distinct contributions of complement factors to platelet activation and fibrin formation in venous thrombus development. Blood.

[5] Nosaka M (2011). Absence of IFN-gamma accelerates thrombus resolution through enhanced MMP-9 and VEGF expression in mice. J Clin Invest.

[6] Luther N (2016). Innate Effector-Memory T-Cell Activation Regulates Post-Thrombotic Vein Wall Inflammation and Thrombus Resolution. Circulation research.

[7] Saghazadeh A, Hafizi S, Rezaei N (2015). Inflammation in venous thromboembolism: Cause or consequence?. Int Immunopharmacol.

[8] von Bruhl ML (2012). Monocytes, neutrophils, and platelets cooperate to initiate and propagate venous thrombosis in mice *in vivo*. J Exp Med.

[9] Wakefield TW (1995). Venous thrombosis-associated inflammation and attenuation with neutralizing antibodies to cytokines and adhesion molecules. Arterioscler Thromb Vasc Biol.

[10] Maldonado-Pena J (2016). Can monocytosis act as an independent variable for predicting deep vein thrombosis?. International journal of cardiology.

[11] Smith RS (2012). Vascular catheters with a nonleaching poly-sulfobetaine surface modification reduce thrombus formation and microbial attachment. Sci Transl Med.

[12] Brill A (2011). von Willebrand factor-mediated platelet adhesion is critical for deep vein thrombosis in mouse models. Blood.

[13] Esmon CT (2008). Crosstalk between inflammation and thrombosis. Maturitas.

[14] Ali T (2006). Monocyte recruitment in venous thrombus resolution. J Vasc Surg.

[15] Henke PK (2004). Deep vein thrombosis resolution is modulated by monocyte CXCR2-mediated activity in a mouse model. Arterioscler Thromb Vasc Biol.

[16] Henke PK (2006). Targeted deletion of CCR2 impairs deep vein thombosis resolution in a mouse model. Journal of immunology.

[17] Humphries J (1999). Monocyte chemotactic protein-1 (MCP-1) accelerates the organization and resolution of venous thrombi. J Vasc Surg.

[18] Dewyer NA (2015). Divergent effects of Tlr9 deletion in experimental late venous thrombosis resolution and vein wall injury. Thromb Haemost.

[19] Soderquest K (2011). Monocytes control natural killer cell differentiation to effector phenotypes. Blood.

[20] Kossmann S (2013). Angiotensin II-Induced Vascular Dysfunction Depends on Interferon-gamma-Driven Immune Cell Recruitment and Mutual Activation of Monocytes and NK-Cells. Arterioscler Thromb Vasc Biol.

[21] Mukhopadhyay S, Antalis TM, Nguyen KP, Hoofnagle MH, Sarkar R (2017). Myeloid p53 regulates macrophage polarization and venous thrombus resolution by inflammatory vascular remodeling in mice. Blood.

[22] Brandt M (2014). Deep vein thrombus formation induced by flow reduction in mice is determined by venous side branches. Clin Hemorheol Microcirc.

[23] Szabo SJ (2000). A novel transcription factor, T-bet, directs Th1 lineage commitment. Cell.

[24] Nahrendorf M (2007). The healing myocardium sequentially mobilizes two monocyte subsets with divergent and complementary functions. J Exp Med.

[25] Hofmann U (2012). Activation of CD4+ T lymphocytes improves wound healing and survival after experimental myocardial infarction in mice. Circulation.

[26] Swirski FK, Nahrendorf M (2013). Leukocyte behavior in atherosclerosis, myocardial infarction, and heart failure. Science.

[27] Nguyen KP (2015). Matrix Metalloproteinase 9 (MMP-9) Regulates Vein Wall Biomechanics in Murine Thrombus Resolution. PLoS One.

[28] Goren I (2009). A transgenic mouse model of inducible macrophage depletion: effects of diphtheria toxin-driven lysozyme M-specific cell lineage ablation on wound inflammatory, angiogenic, and contractive processes. Am J Pathol.

[29] Wenzel P (2011). Lysozyme M-Positive Monocytes Mediate Angiotensin II-Induced Arterial Hypertension and Vascular Dysfunction. Circulation.

[30] Frantz S (2013). Monocytes/macrophages prevent healing defects and left ventricular thrombus formation after myocardial infarction. FASEB J.

[31] Meng XM (2016). Inflammatory macrophages can transdifferentiate into myofibroblasts during renal fibrosis. Cell death & disease.

[32] Siefert SA (2014). Enhanced venous thrombus resolution in plasminogen activator inhibitor type-2 deficient mice. J Thromb Haemost.

[33] Kosaka H, Yoshimoto T, Yoshimoto T, Fujimoto J, Nakanishi K (2008). Interferon-gamma is a therapeutic target molecule for prevention of postoperative adhesion formation. Nat Med.

[34] Kawano Y, Matsui N, Kamihigashi S, Narahara H, Miyakawa I (2000). Effects of interferon-gamma on secretion of vascular endothelial growth factor by endometrial stromal cells. American journal of reproductive immunology.

[35] Trompezinski S, Denis A, Vinche A, Schmitt D, Viac J (2002). IL-4 and interferon-gamma differentially modulate vascular endothelial growth factor release from normal human keratinocytes and fibroblasts. Exp Dermatol.

[36] Demyanets S (2011). Oncostatin M-enhanced vascular endothelial growth factor expression in human vascular smooth muscle cells involves PI3K-, p38 MAPK-, Erk1/2- and STAT1/STAT3-dependent pathways and is attenuated by interferon-gamma. Basic Res Cardiol.

[37] Ensan S (2016). Self-renewing resident arterial macrophages arise from embryonic CX3CR1(+) precursors and circulating monocytes immediately after birth. Nature immunology.

[38] Sato N (1990). Actions of TNF and IFN-gamma on angiogenesis *in vitro*. The Journal of investigative dermatology.

[39] Kearon C (2001). Natural history of venous thromboembolism. Semin Vasc Med.

[40] Ozcan Cetin EH (2017). Platelet-to-lymphocyte ratio as a novel marker of in-hospital and long-term adverse outcomes among patients with acute pulmonary embolism: A single center large-scale study. Thromb Res.

[41] Yang M (2016). The role of mononuclear cell tissue factor and inflammatory cytokines in patients with chronic thromboembolic pulmonary hypertension. J Thromb Thrombolysis.

[42] Aldabbous L (2016). Neutrophil Extracellular Traps Promote Angiogenesis: Evidence From Vascular Pathology in Pulmonary Hypertension. Arterioscler Thromb Vasc Biol.

[43] Libby P (2002). Inflammation in atherosclerosis. Nature.

[44] Hulsmans M, Sam F, Nahrendorf M (2016). Monocyte and macrophage contributions to cardiac remodeling. J Mol Cell Cardiol.

[45] Ridker PM (2018). Relationship of C-reactive protein reduction to cardiovascular event reduction following treatment with canakinumab: a secondary analysis from the CANTOS randomised controlled trial. Lancet.

[46] Finotto S (2002). Development of spontaneous airway changes consistent with human asthma in mice lacking T-bet. Science.

[47] Clausen BE, Burkhardt C, Reith W, Renkawitz R, Forster I (1999). Conditional gene targeting in macrophages and granulocytes using LysMcre mice. Transgenic Res.

[48] Buch T (2005). A Cre-inducible diphtheria toxin receptor mediates cell lineage ablation after toxin administration. Nat Methods.

[49] Diaz JA, Farris DM, Wrobleski SK, Myers DD, Wakefield TW (2015). Inferior vena cava branch variations in C57BL/6 mice have an impact on thrombus size in an IVC ligation (stasis) model. J Thromb Haemost.

[50] Chomczynski P, Sacchi N (1987). Single-step method of RNA isolation by acid guanidinium thiocyanate-phenol-chloroform extraction. Anal Biochem.

[51] Towers B (1953). A Modification of Masson Trichrome Stain Which Differentiates in Colour between Striated and Smooth Muscle. J Physiol-London.

